# Proceedings of the Buffalo Medical and Surgical Association

**Published:** 1889-02

**Authors:** Wm. A. Hoddick

**Affiliations:** Secretary pro tem.


### *PROCEEDINGS OF THE BUFFALO MEDICAL AND SURGICAL ASSOCIATION.*

Reported by Wm. A. Hoddick, M. D., Secretary *pro tem.*

Regular monthly meeting held in the Mechanics' Institute Building, Tuesday evening, December 4, 1888.

The President, Dr. P. W. VanPeyma, in the chair.

Present—Drs. Mynter, Park, Hayd, Wheeler, F. H. Potter, W. W. Potter, Banta, Falk, Flagg, Wetmore, Renner, Brecht, Tremaine, Morse, Rogers, Hubbell, Coakley, Mann, Rochester, Ring, Niemand, Schade and Callahan.

Drs. Rogers and Persons were elected members of the Association.

Dr. Morse, Post-Surgeon at Fort Porter, was invited to be present at the meetings, and to participate in the discussions. He thanked the Association for the compliment, and expressed much anticipated pleasure and benefit, that he would appreciate, in accepting the invitation.

Dr. F. H. Potter then read a paper on the "Treatment of Acute Coryza" (see page 302, January number).

Dr. Park read a paper on the "Radical Cure of Hernia." After rehearsing the numerous methods of treatment that have been proposed, he took up the modern surgical procedure which has been advocated by various operators. He favored Czerney's method of ligating the sac as high up as possible, exsecting the part below the ligature in all cases except congenital herniæ. In these the lower portion is left and closed by suture or ligature, so as to form a tunica vaginalis for the testicle, the part between the ligature being removed. Beginning from below, the pillars of the ring are brought together with wire sutures, leaving a small opening at the bottom for the cord. The author at first made a shoe-lace suture, but found it difficult to get accurate approximation of the pillars of the ring. Catgut was at first used for suturing, but was discarded later for silk. At present he employs silver wire sutures, a sinus having formed in one of his cases with the throwing off of one of the silk sutures. The incision should be long enough, great care being taken to separate the sac from the cord. Intestines are replaced into the abdomen, and the sac ligated as high up as possible. The silver sutures are twisted, the cut end being turned in and left in place. Femoral and umbilical herniæ are to be similarly treated, extirpation of the sac being essential. "In strangulated hernia, after relieving the strangulation, proceed in the same way. Do not allow the wearing of a truss after the operation." The author has operated in forty cases, uncomplicated by strangulation or otherwise, with universal success. The patients were of all ages, from five years to sixty. Six of the operations were for umbilical, and eight for femoral hernia. In one of the cases of umbilical hernia, a fat woman, in whom the tumor hung down to the thighs, there existed a number of tumors within the sac, together with ulcerations on the surface. Fearing the tumors to be malignant, he had at first declined to operate, but, the patient assuming all risks, he operated, removed the sac and tumors, closing the wound with three layers of sutures, including the (1) peritoneum, (2) the deep and (3) superficial tissues. Adherent pieces of omentum are to be loosened and appendices of the same are to be securely ligated and cut off, especially if they cannot be easily replaced into the abdomen. Dr. Park takes the position that hernia, with its attendant liability to strangulation, is more dangerous to life than the operation for its cure, and, therefore, recommends it in all cases.

DISCUSSION.

Dr. Wheeler did not think that in acute coryza we should exclude fresh air from the sleeping apartments, and that pure air, even if night air, was beneficial. He suspected diet to be the frequent cause of the affection, and had observed that a brisk cathartic often hastened recovery. He was deeply interested in the subject of Dr. Park's paper. He had performed Heaton's operation in twelve cases, all of which failed to cure; hence he had given up the operation. Has operated lately according to McBurney's method, leaving the wound to heal by granulation. He unites the conjoined tendon to the skin, and fastens the stump of the sac into the upper angle of the ring. The large cicatricial plug thus formed, he thought, effectually closed the ring.

Dr. Mynter agreed with Dr. Park as to the advisability of the operation. The two necessary adjuncts to success are, first, the extirpation of the sac, and second, the proper closure of the ring. He thought Ball's method of torsion of the sac of advantage in femoral hernia. He operates after the Czerney method, and at first used catgut sutures, but now employs silver wire. In one case there was slight suppuration, with throwing off of one of the wire sutures. He had operated on sixty cases of strangulated hernia with success. He thinks it most important to remove the sac.

Dr. W. W. Potter gave the history of a case of strangulated umbilical hernia, in which he had made laparatomy, part of the contents of which proved to be portion of one lobe of the liver. The patient had been suffering from severe biliary vomiting, which was relieved after the laparatomy, and the successful cure of the hernia, a large one, was prompt.

Dr. Wetmore presented a pathological specimen, the contents of a double hernia. The patient, aged sixty-one, with an irreduceable hernia, was operated upon by himself and Dr. Boswell. Both hernias were on the right side; the larger direct and opposite the internal inguinal ring, the smaller at the external ring. The contents of the larger tumor proved to be mainly omentum. The smaller tumor contained what he supposed to be an ovary. The patient made a good recovery. Dr. Wetmore had operated frequently after the method of Heaton, with but few failures and nine radical cures. Has not operated in strangulated hernia, as he has always succeeded in reducing them.

Dr. MANN, while not having had experience in the treatment of hernia, thought that any measure that depended on a plug of cicatricial or other tissue for support, would prove unsuccessful. After laparatomy, it was always the cicatrix left after the removal of the drainage-tube which proved the weakest spot and permitted hernia to protrude. On this account he dreads the use of the drainage-tube, but prefers to suture, first, the peritoneum; second, the fascia; and third, the skin.

Dr. COAKLEY verified Dr. Potter's statement regarding the efficacy of hot baths with quinia or atropia in acute coryza. While many cases were due to changes of temperature, he thought that more frequently the secretions of the bowels were at fault. He consequently gave a cathartic to restore the equilibrium of the circulation.

Dr. POTTER, in closing the discussion, said that in excluding night air, he did not mean that ventilation should be neglected. The Scandinavians rigorously exclude night air from their sleeping apartments, and coryza is almost unknown among them. Birds and animals warm the air they breath by huddling together and placing the nostrils close to the warm skin. There should be no great variation in temperature between the living and sleeping apartments.

Dr. PARK agreed with Dr. Mynter that the success of the operation depended on obliteration of the sac. Heaton's method was unscientific. The position of the sac was uncertain, and the needle enters in a blind hit-or-miss sort of a way. Umbilical herniæ he had found most frequent in stout women. The hernial ring itself is generally small, but the tumor may spread out indefinitely between the layers of the abdominal fascia.

No voluntary contributions or reports.

Miscellaneous business.

The next meeting of the Association was postponed to Tuesday, January 8, 1889.

Essayists for next meeting were announced: Dr. Ernest Wende, subject, "Skin Diseases;" Dr. W. C. Phelps, subject, "Surgery of the Knee Joint."

[NOTE.—These minutes were not received in time to appear in the January number of the JOURNAL.—ED.]

